# MiR‐21‐5p/dual‐specificity phosphatase 8 signalling mediates the anti‐inflammatory effect of haem oxygenase‐1 in aged intracerebral haemorrhage rats

**DOI:** 10.1111/acel.13022

**Published:** 2019-08-10

**Authors:** Yetong Ouyang, Dongling Li, Han Wang, Zhigang Wan, Qinghua Luo, Yuqin Zhong, Min Yin, Zhengfang Qing, Zhengyu Li, Bing Bao, Zhiying Chen, Xiaoping Yin, Ling‐Qiang Zhu

**Affiliations:** ^1^ Department of Neurology Affiliated Hospital of Jiujiang University Jiujiang China; ^2^ Center for Clinical Precision Medicine Jiujiang University Jiujiang China; ^3^ Department of Neurology Second Affiliated Hospital of Nanchang University Nanchang China; ^4^ Department of Pathophysiology, Tongji Medical College Huazhong University of Science and Technology Wuhan China

**Keywords:** aging, dual‐specificity phosphatase 8, haem oxygenase‐1, intracerebral haemorrhage, microRNA

## Abstract

Intracerebral haemorrhage (ICH) is a severe neurological disorder caused by bleeding within the brain tissue. Inflammation has been implicated in ICH pathogenesis and is a potential therapeutic target for ICH. Haemin, an activator of haem oxygenase‐1 (HO‐1), rapidly increases HO‐1 protein expression and activity and has been shown to distinctly affect anti‐inflammatory functions after central nervous system (CNS) injury. However, less is known about the mechanisms that underlie the anti‐inflammatory effects of haemin in aged rats post‐ICH. Here, we performed microarray analysis to identify miRNAs that respond strongly to HO‐1 regulation in ICH rats and found that miR‐21‐5p induced the most significant change. Using Kyoto Encyclopedia of Genes and Genomes (KEGG) enrichment and Gene Ontology (GO) analysis, we focused on dual‐specificity phosphatase 8 (DUSP8) from the predicted miR‐21‐5p targets. Luciferase reporter assays confirmed that miR‐21‐5p bound directly to DUSP8. MiR‐21‐5p upregulation in vitro downregulated DUSP8 expression. Importantly, intracerebroventricularly injecting antagomir for miR‐21‐5p (A‐miR‐21‐5p), which was used to inhibit miR‐21‐5p in aged ICH rats, significantly reduced the neurological defects, repaired cognitive impairment, alleviated blood–brain barrier (BBB) permeability, inhibited neuronal apoptosis posthaemorrhage and accelerated haematoma absorption. In addition, serum miR‐21‐5p levels were notably elevated in patients relative to healthy individuals and were correlated with National Institutes of Health Stroke Scale (NIHSS) scores and clinical outcomes. In summary, A‐miR‐21‐5p increased HO‐1 expression in cerebral haematomas, thus eliciting the DUSP8‐modulated perifocal neuroprotective effect of haemin. MiR‐21‐5p with haemin therapy may be a potential therapy post‐ICH.

## 1 INTRODUCTION

With aging, spontaneous intracerebral haemorrhage (ICH) is a devastating form of stroke with a 30%–50% mortality rate in elderly patients (Tarantini et al., 2017). More than half of ICH survivors exhibit left haemiplegia, speech disorders and other vascular cognitive impairments for which no effective therapy is available (Zhao et al., 2017; Chen‐Roetling et al., 2017). Recent evidence suggests that stroke causes an inflammatory cascade that accelerates oedema formation around haematomas, exacerbates the mass effect and amplifies the cellular apoptotic range (Ren et al., 2018). Haem oxygenase‐1 (HO‐1) catalyses the breakdown of haem into bilirubin, ferrous iron and carbon monoxide and is a potential therapeutic target for ICH (Chen‐Roetling et al., 2017).

HO‐1 signal activation exerts a protective effect by preventing oxidative stress damage by accelerating catabolism of pro‐oxidant haem and oxygen‐free radicals (Lin et al., 2015). HO‐1 is expressed at low physiological levels, which can be increased via external stimulation, such as hypoxia, oxidative stress, inflammatory reactions, haem, endotoxins and heavy metals (Even et al., 2018). Our group found that increased HO‐1 expression begins 6 hr after ICH and that this change is consistent with the peak time of brain oedema (Yin, Chen, Zhou, Wu, & Bao, 2015). In recent years, application of the HO‐1 activator, haemin, has made rapid progress in food and medicine, especially in treating iron deficiency anaemia. The Food and Drug Administration (FDA) approved intravenous haemin for treating acute intermittent porphyria (1–4 mg kg^−1^ day^−1^ for 3–14 days) (Forbes et al., 1980; Prout et al., 1988; Tripathi & Singh, 2016). However, the underlying mechanisms for HO‐1 protection remain unclear.

As novel biomarkers and regulatory small molecules, miRNAs play important roles in diagnosing and treating several diseases (Chen et al., 2016; Wang et al., 2018). The mechanism lies in binding the 3′ untranslated region (3′UTR) of the target gene. Accumulating evidence has linked miRNA dysregulation to the pathological process of cerebral haemorrhaging. For example, we previously reported that applying miR‐130a inhibitors in ICH rats reduced brain oedema and blood–brain barrier (BBB) permeability, and increased neurological deficit scores, while miR‐130a mimics increased monolayer permeability (Wang et al., 2016). Upregulated miR‐23a‐3p in ICH patients produced anti‐inflammatory and antioxidative stress effects by suppressing zonula occludens‐1 (ZO‐1) (Hu, Wang, Huang, Wang, & Zhang, 2018). Furthermore, inhibiting miR‐27b can alleviate oxidative stress in the cerebral cortex (Guo et al., 2013). However, whether miRNAs are involved in protecting HO‐1 activation remains unclear.

In the present study, we first performed microarray and qPCR analysis and identified that miR‐21‐5p is the miRNA that responds most significantly to HO‐1 regulation. We also validated that dual‐specificity phosphatase 8 (DUSP8) is the direct target of miR‐21‐5p. Lateral ventricular injection of A‐miR‐21‐5p in aging ICH rats inhibited miR‐21‐5p; alleviated behavioural deficits, cerebral oedema, BBB permeability and neuronal apoptotic neuroinflammation; and promoted haematoma absorption in the perihaematomal area. Furthermore, serum samples from older patients with ICH showed that miR‐21‐5p levels were distinctly elevated compared with those in the patients' healthy peers, indicating the potential of miR‐21‐5p for predicting neurological deficit and clinical outcome severity.

## 2 RESULTS

### 2.1 miRNA expression profiles upon HO‐1 activation

**Figure 1:**
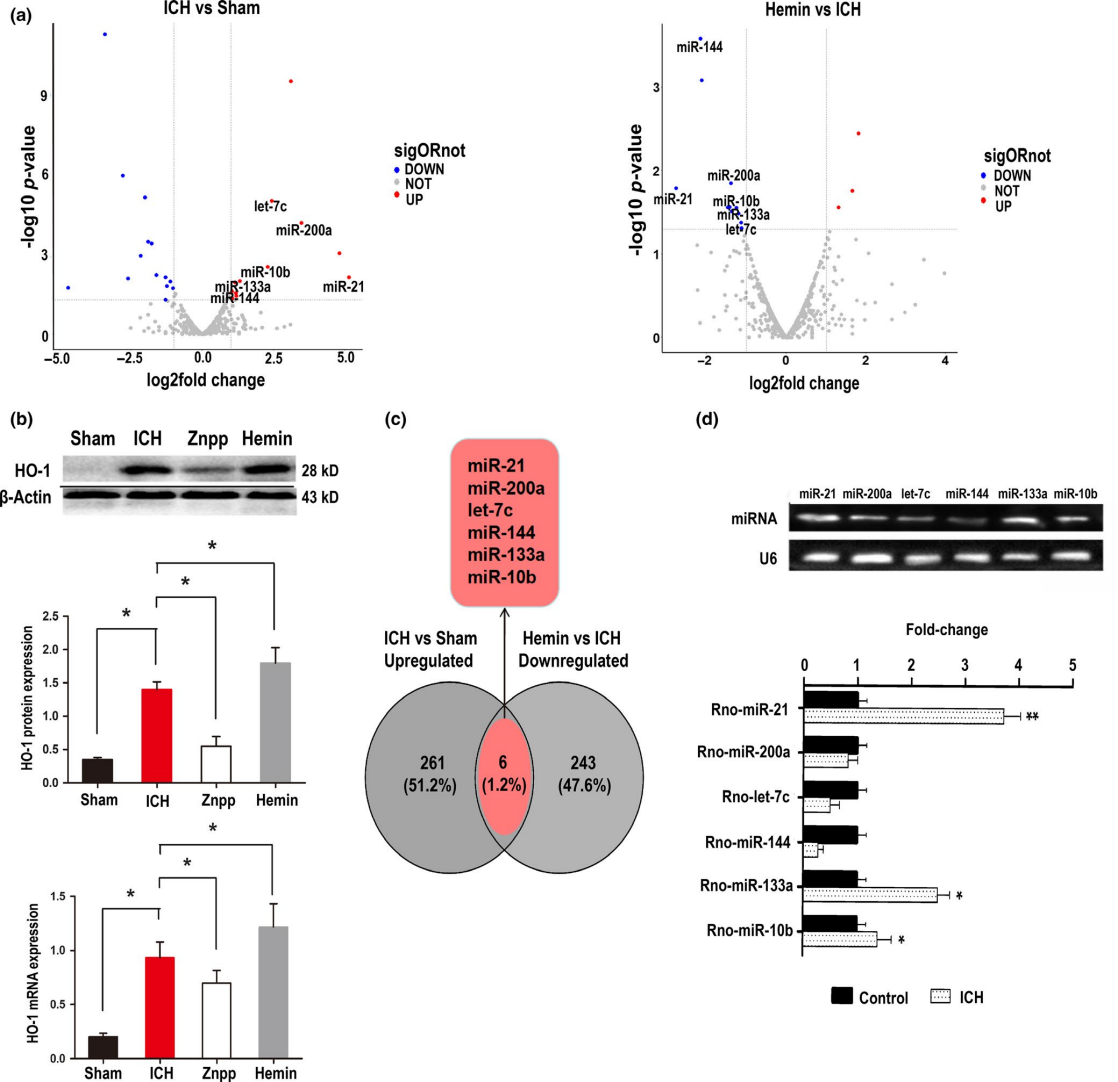
Expression profiles of miRNAs upon HO‐1 activation. (a) The Volcano plot compiled differentially expressed miRNAs between the two groups (ICH vs. Sham and haemin vs. ICH). The red dots indicate upregulated miRNAs, and the blue dots indicate downregulated miRNAs (*p* < 0.05). (b) HO‐1 mRNA and protein level modulation via haemin and ZnPP treatment in ICH‐model rats was confirmed by Western blot and PCR. Data are the means ± *SEM* for 3 independent experiments. *p < 0.05. (c) Venn diagram of miRNAs expressed in two groups as determined by microarray sequencing, differentially expressed miRNAs that downregulated ICH versus haemin merged with upregulated ICH versus sham = 6. (d) For each miRNA candidate, qRT–PCR measurements were performed to obtain a mean CT value for each sample. CT values of the different samples were compared using the 2^−ΔΔCT^ method with U6 expression levels used as an internal reference. All results are presented as the means ± *SEM* of three independent experiments. *p < 0.05, upregulation versus control

We first performed miRNA microarray analysis to find potential candidate miRNAs involved in protecting HO‐1. Among the 2,668 miRNAs detected (Figure S1a), we found 267 upregulated and 349 downregulated miRNAs between the ICH and sham groups and 370 upregulated and 246 downregulated miRNAs between the haemin and ICH groups (Figure 1a). HO‐1 expression was elevated 24 hr after ICH with haemin therapy but rapidly decreased with ZnPP (HO‐1 inhibitor) therapy (Figure 1b). Of the 616 aberrantly expressed miRNAs in the ICH versus sham samples, 12 were upregulated, and 14 were downregulated (log_2_ fold change > 1.5, *p* < 0.05). In the haemin versus ICH group, 3 miRNAs were significantly upregulated, and 11 were significantly downregulated. Venn diagrams indicated that 6 intersected miRNAs (miR‐144‐3p, miR‐200a‐3p, let‐7c‐5p, miR‐133a‐5p, miR‐10b‐5p and miR‐21‐5p) were upregulated in ICH rats but downregulated after haemin treatment (Figure 1c). Further qRT–PCR validation suggested that miR‐21‐5p was the most significantly upregulated miRNA in ICH rats (Figure 1d) and was thus selected for further investigation.

We next performed a bioinformatic analysis to seek the molecular targets of miR‐21‐5p. The TargetScan (http://www.targetscan.org), PITA (http://genie.weizmann.ac.il/pubs/mir07/mir07_data.html) and MiRanda (http://www.microrna.org/microrna/home.do) databases were used to predict miR‐21‐5p target genes. We further investigated the top 10 ranked pathways after analysing the Kyoto Encyclopedia of Genes and Genomes (KEGG) pathway enrichment (Figure S2a) of 220 target genes (Table S1). For this analysis, we set the standard to *p* < 0.05 as a significant Gene Ontology (GO) enrichment condition. The details of these GO functions are listed in the Supplementary Material (Figure S2b). The downregulated genes in the nervous system contained two biological process (BP) categories: commissural neuron axon guidance (GO: 0071679, *p* = 0.077) and negative regulation of neuron apoptotic processes (GO: 0043524, *p* = 0.006).

Among the predicted target genes (Table S1), the highest ranking gene, YOD1 deubiquitinase (YOD1), showed little association with the inflammatory response (Kim & Jho, 2017). However, the second ranking gene, dual‐specificity phosphatase 8 (DUSP8), was strongly relevant to the top KEGG enrichment signalling pathway, the MAPK signalling pathway, which is an inflammatory and immune response pathway (Okami et al., 2013; Liu et al., 2016; Oehrl, Cotsiki, & Panayotou, 2013). Therefore, DUSP8 was selected for further study as the target gene related to inflammatory miR‐21‐5p.

### 2.2 DUSP8 is the direct target of miR‐21‐5p

**Figure 2:**
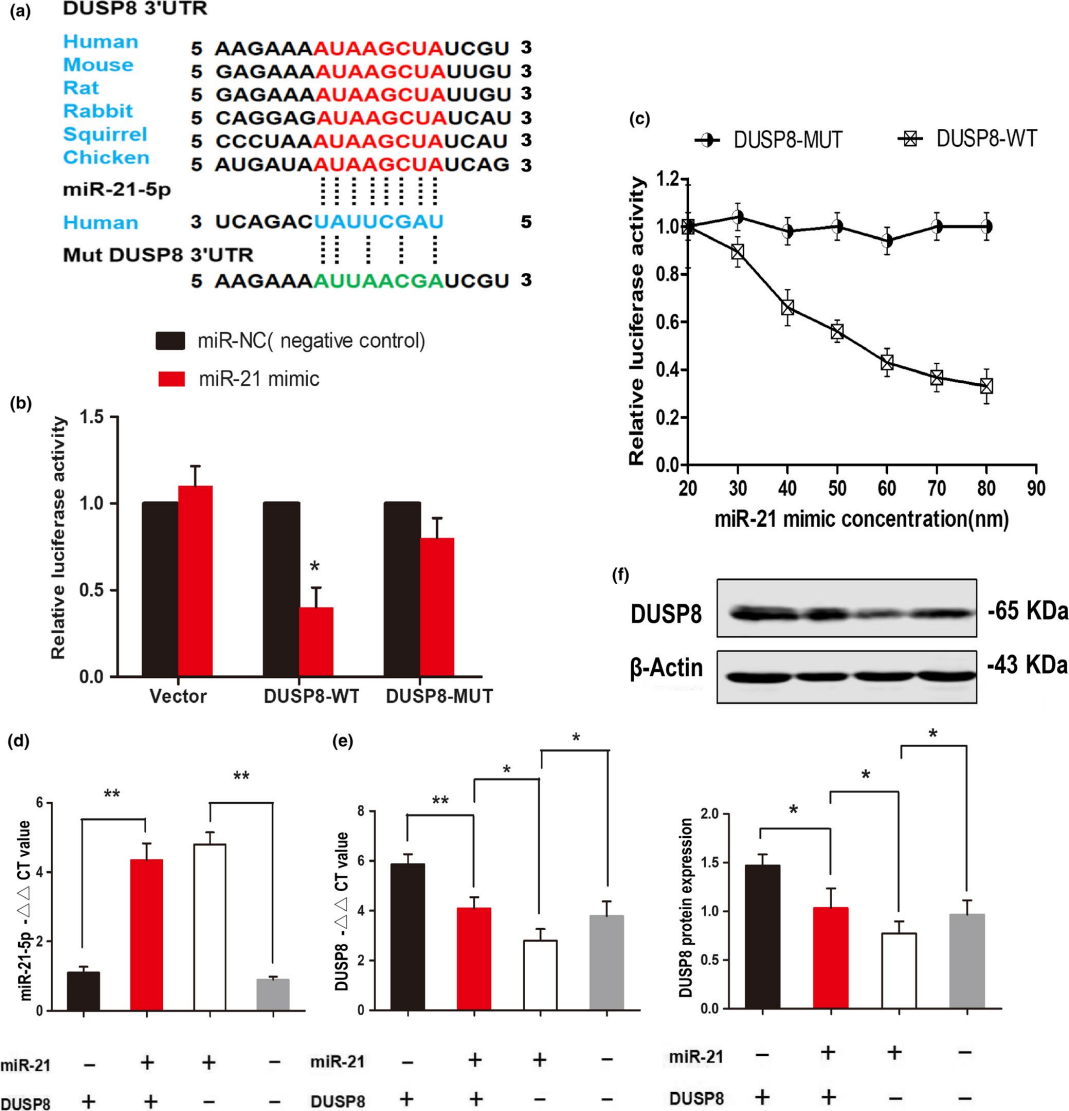
DUSP8 is the direct target of miR‐21‐5p. (a) Binding site prediction in the 3′UTR of DUSP8 on mammalian miR‐21‐5p by bioinformatics analysis. To generate the firefly luciferase reporter plasmids, pmirGLO containing the entire 3'UTR mutant of DUSP8 (DUSP8‐MUT) or pmirGLO containing the entire 3'UTR of DUSP8 (DUSP8‐WT) was transiently transfected into HEK293 cells. (b) Bar graph showing relative luciferase activities in HEK293T cells cotransfected with the miR‐21 mimic (50 nM) or mimic control (miR‐NC, 50 nM) plus pmirGLO‐DUSP8‐WT (DUSP8‐WT) or pmirGLO‐DUSP8‐MUT (DUSP8‐MUT) or vector. (c) Line graph summarizing the relative luciferase reporter activities with increasing miR‐21 concentrations. Data are the means ± *SEM* of 3 independent experiments. *p < 0.05. (d–f) Relative expressions were measured after 48 hr. Relative expression of miR‐21 (d), DUSP8 mRNA (e) and DUSP8 protein (f) in HEK293T cells transfected with miR‐NC + DUSP8, miR‐21 + DUSP8, miR‐21 + Vector or miR‐NC + Vector. Data are the means ± *SEM* of 3 independent experiments for both parameters. *p < 0.05

Since the putative binding site for the 3′UTR of DUSP8 to miR‐21‐5p is conserved among mammalians (Figure 2a), we constructed luciferase reporter plasmids, pmirGLO containing the wild‐type (pmirGLO‐DUSP8‐WT) or mutant (pmirGLO‐DUSP8‐MUT) 3′UTR of DUSP8 with the luciferase gene. No difference in luciferase activity was found between cells transfected with the control vector and those transfected with the vector containing the 3′UTR mutant of DUSP8. In contrast, overexpressing miR‐21‐5p in cells transfected with the entire WT‐DUSP8 3'UTR reduced the luciferase activity by more than 60% (Figure 2b). We also overexpressed miR‐21‐5p using different miR‐21‐5p mimic concentrations. As the miR‐21‐5p concentration increased, the luciferase reporter activity in the WT‐DUSP8 3′UTR continuously decreased (Figure 2c). To further verify the targeted suppressed interaction between miR‐21‐5p and DUSP8, we transfected HEK293T cells with the plasmids, miR‐NC + DUSP8, miR‐21 + DUSP8, miR‐21 + Vector or miR‐NC + Vector. RT–PCR and Western blot analysis showed that miR‐21‐5p upregulation (Figure 2d) in HEK293T cells resulted in downregulated DUSP8 expression (Figure 2e,f). This finding supports the hypothesis that miR‐21‐5p targets the DUSP8 3'UTR.

### 2.3 DUSP8‐induced activation of the p‐ERK/HO‐1 pathway alleviated haemorrhagic injuries by inhibiting miR‐21‐5p

**Figure 3:**
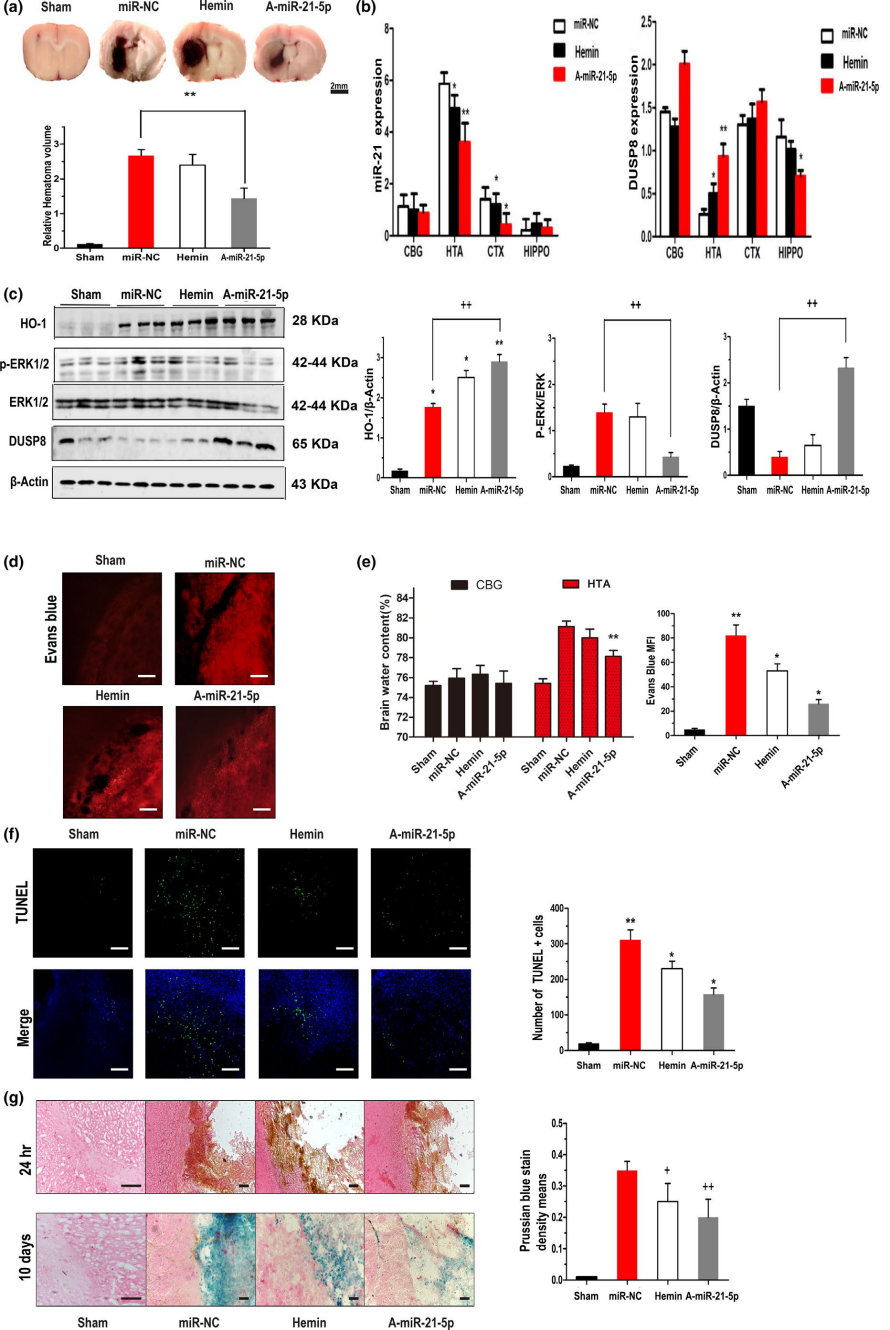
Inhibition of miR‐21‐5p alleviates haemorrhagic injury. (a) Representative ICH brain section images and quantitative data on lesion volumes at 24 hr post‐ICH with different dispositions. Scale bar = 2 mm. The bar graph shows the volumes of total haematoma in the ipsilateral hemisphere normalized to the total volumes of the contralateral hemisphere. Data are the means ± *SEM* of six rats per group. **p < 0.01. (b) Modulation of miR‐21 and DUSP8 mRNA levels was confirmed by qRT–PCR between different brain areas (CBG: contralateral basal ganglia; HTA: haematoma; CTX: cerebral cortex; HIPPO: hippocampus) 24 hr post‐ICH. Data are the means ± *SEM* of 3 independent experiments for all 4 parameters. *p < 0.05 by Student's *t* test. (c) Levels of HO‐1, DUSP8, p‐ERK and ERK in perihaematomal tissues of ICH rats were determined by Western blot 24 hr after ICH. β‐actin served as an internal control. (d) Evans blue (EB) extravasation 24 hr post‐ICH. Scale bar = 100 μm. (e) Brain water content of rats 24 hr post‐ICH (CBG: contralateral basal ganglia; HTA: haematoma). (f) TUNEL staining of apoptotic cells in the brain sections. The representative images of perihaematomal areas are presented. Scale bars, 100 μm. Bar graph shows the number of TUNEL + cells in four groups. **p < 0.01 versus sham group. *p < 0.05 versus miR‐NC group. (g) Prussian blue‐stained section (counterstained with haematoxylin and eosin) shows the location of iron deposits in the perilesional areas on postoperative day 10. Scale bar = 100 m, four groups share scale bars. ++*p* < 0.01 versus miR‐NC group. +*p* < 0.05 versus miR‐NC group

Because miR‐21‐5p binds to the DUSP8 3′UTR to function as a putative activator of the HO‐1 anti‐inflammatory pathway, we further explored this relationship by administering haemin and antagomir of miR‐21‐5p (A‐miR‐21‐5p) to collagenase‐induced rats as previously described. Rats that received haemin treatment presented a haemorrhagic volume equal to that of the miR‐NC group, but the A‐miR‐21‐5p‐treated group presented a haemorrhagic injury volume that was reduced by 43% of that of the miR‐NC group at 24 hr post‐ICH (*p* = 0.0012; Figure 3a). To determine whether administering A‐miR‐21‐5p or haemin affected miR‐21‐5p and DUSP8 expression in different brain areas after 24 hr, we performed qRT–PCR and found that miR‐21‐5p was upregulated, while DUSP8 was downregulated compared with haemin in haematomas injected with A‐miR‐21‐5p. No distinct differences were observed among the other three groups (Figure 3b). By using web‐STRING analysis showing a protein–protein interaction network of DUSP8, we found that MAPK1, also known as ERK2, was closely related to DUSP8 via the interaction network (Figure S2c). Next, we evaluated the effect on MAPK phosphorylation. The phosphorylation levels of ERK1/2 and the putative DUSP8 responsible for phosphorylating neurons in the area of the inflammatory injury were analysed via Western blot. Knockdown of miR‐21‐5p significantly upregulated DUSP8 expression (*p* < 0.01; Figure 3c), reduced phosphorylated ERK1/2 (*p* < 0.01; Figure 3c) and increased HO‐1 levels (*p* < 0.05; Figure 3c) surrounding the haematoma. Thus, A‐miR‐21‐5p indirectly reversed ICH‐induced ERK1/2 phosphorylation and augmented activation of the HO‐1 anti‐inflammatory pathway.

### 2.4 Knockdown of miR‐21‐5p alleviated haemorrhagic inflammatory injury 24 hr post‐ICH

Striatal water content increased severely within 24 hr in the haemorrhagic hemisphere of the brain. However, no differences in brain water content were observed in the contralateral basal ganglia. The water content was prominently reduced via A‐miR‐21‐5p treatment rather than haemin (Figure 3e). Evans blue extravasation of the miR‐NC group was significantly higher than that of the sham group (Figure 3d), suggesting increased BBB permeability after ICH. In contrast, A‐miR‐21‐5p remarkably inhibited neurovascular permeability after ICH and produced less Evans blue extravasation than haemin. These results demonstrated that A‐miR‐21‐5p better preserved BBB integrity and function than haemin after inducing ICH. TUNEL staining showed a high positive‐cell density in the area of inflammatory lesions around the haematoma (Figure 3f). Statistical analysis showed that the rate of apoptotic cells increased with ICH (*p* < 0.01), while A‐miR‐21‐5p injection diminished cell death around the haematoma (*p* < 0.05), implying that miR‐21‐5p inhibition attenuated neuronal apoptosis after haemorrhage.

### 2.5 Knockdown of miR‐21‐5p promoted haematoma absorption 10 days post‐ICH

Previous studies suggest that the haematoma absorption period peaks between 10 and 14 days after cerebral haemorrhage (Moussouttas et al., 2010). To assess tissue iron deposition after cerebral haemorrhage, we used Prussian blue staining, which displays ferric iron deposits predominantly in phagocytes or the extracellular interstitium (Figure 3g). In the acute ICH phase, we found no iron deposition in the basal ganglia. The deepest degree of staining of the miR‐NC group was observed 10 days after inducing ICH, while haemin and A‐miR‐21‐5p reversed the aggravating trend of pathological iron deposition changes in the brain tissue. Similarly, the A‐miR‐21‐5p group showed obviously weaker staining of the haematoma pathophysiology than did the haemin group. We speculate that this may have been related to haematoma absorption in the vascular rupture wound‐healing process.

### 2.6 Inhibiting miR‐21‐5p attenuated neuroinflammation

We examined the neuroinflammation by analysing the Iba‐1‐positive microglial numbers and inflammatory factor content (Tokizane et al., 2017). The Iba‐1‐positive activated microglia were increased in the miR‐NC‐treated group 24 hr post‐ICH (Figure S3a). Both haemin and A‐miR‐21‐5p treatment significantly reduced microglial activation (Figure S3b). Furthermore, haemin and A‐miR‐21‐5p suppressed inflammatory factors, including IL‐1β, IL‐6 and TNF‐α (Figure S4). Thus, inhibiting miR‐21‐5p ameliorated the neuroinflammation in ICH.

### 2.7 Inhibiting miR‐21‐5p restored the neurological functions

**Figure 4:**
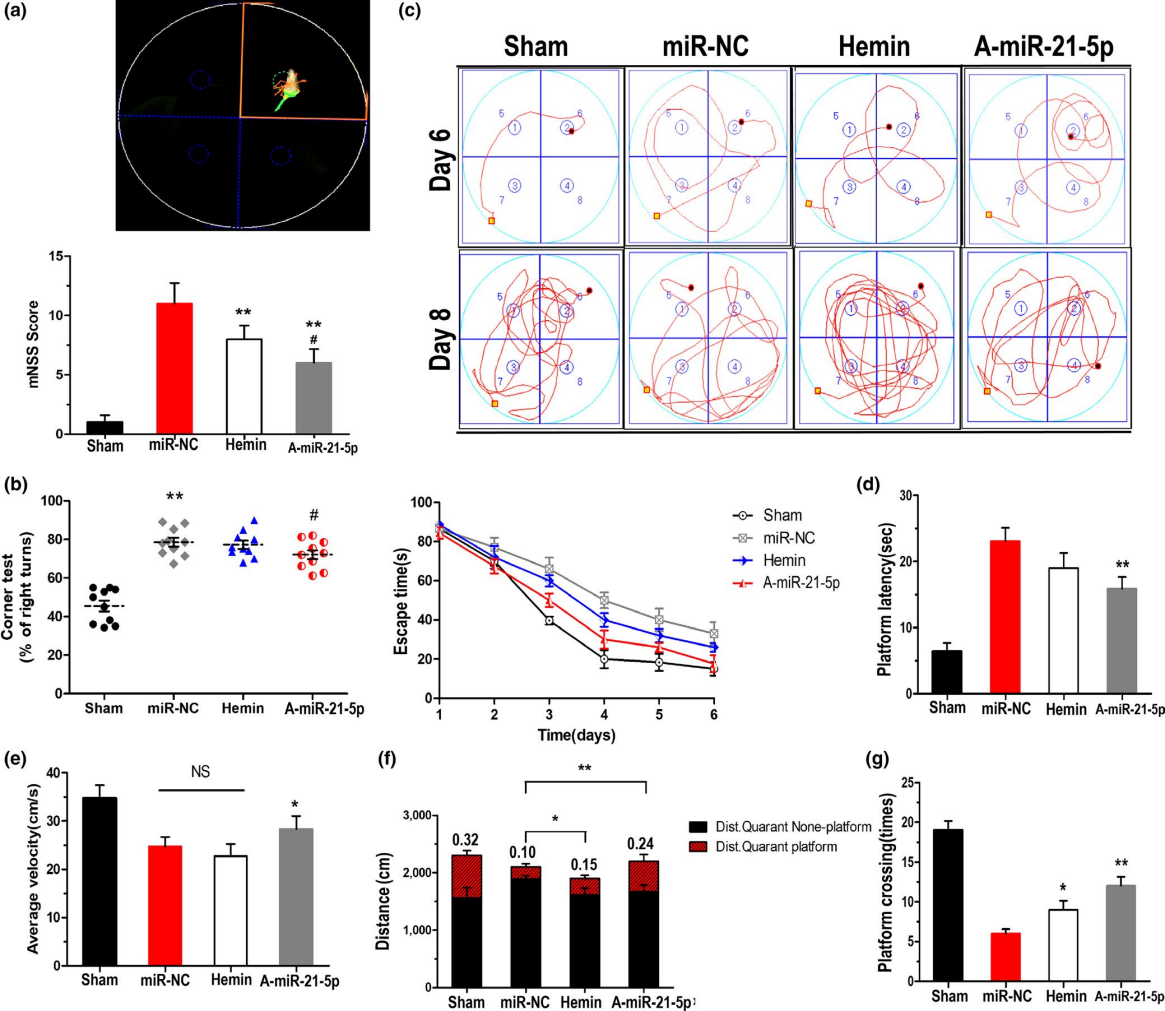
Inhibition of miR‐21‐5p restores neurological functions. (a,b) The mNSS (a) and corner test (b) were assessed 24 hr post‐ICH to assess neurological deficits in rats. For hidden platform training, the line graph (c) shows the escape time to find the hidden platform. The bar graph (d) shows the platform latency to reach the hidden platform. For the probe trial test, the bar graph shows (f) the ratio of distance spent in the target quadrant platform to total distance, the time spent crossing the platform (g) and the average velocity (e) of the four groups. Data represent the means ± *SEM* of 15 rats per group. *p < 0.05 by Student's *t* test

Neurological deficits were performed 24 hr after ICH induction. The miR‐NC‐treated ICH rats had significantly more severe neurological deficits than did the sham‐surgery rats (Figure 4a). Consistent with prior observations (MacLellan et al., 2008), A‐miR‐21‐5p injection produced fewer neurological deficits (mNSS) than did the haemin treatment. In the corner test, the frequency of right turns in the ICH group increased markedly after haemorrhagic damage compared with those of the sham group, while A‐miR‐21‐5p attenuated the frequency of right turns. Haemin therapy showed no significant effect (Figure 4b).

We further evaluated the learning and memory abilities in the miR‐21‐5p‐treated ICH rats by using a Morris water maze. Figure 4c shows each group's escape time tendency in the hidden platform training. The A‐miR‐21‐5p group had the shortest escape latency (time from the starting point to the centre of the target quadrant; Figure 4d) and the fastest swimming velocity (Figure 4e). When the platform was removed, swimming distances (Figure 4f) and crossing times (Figure 4g) were longer in the target quadrant, while the haemin treatment also increased the crossing time frequencies and swimming distances in the target quadrant. The miR‐NC group differed from the sham group but not significantly.

### 2.8 Serum miR‐21‐5p was elevated in elderly ICH patients and was associated with clinical outcomes

**Figure 5:**
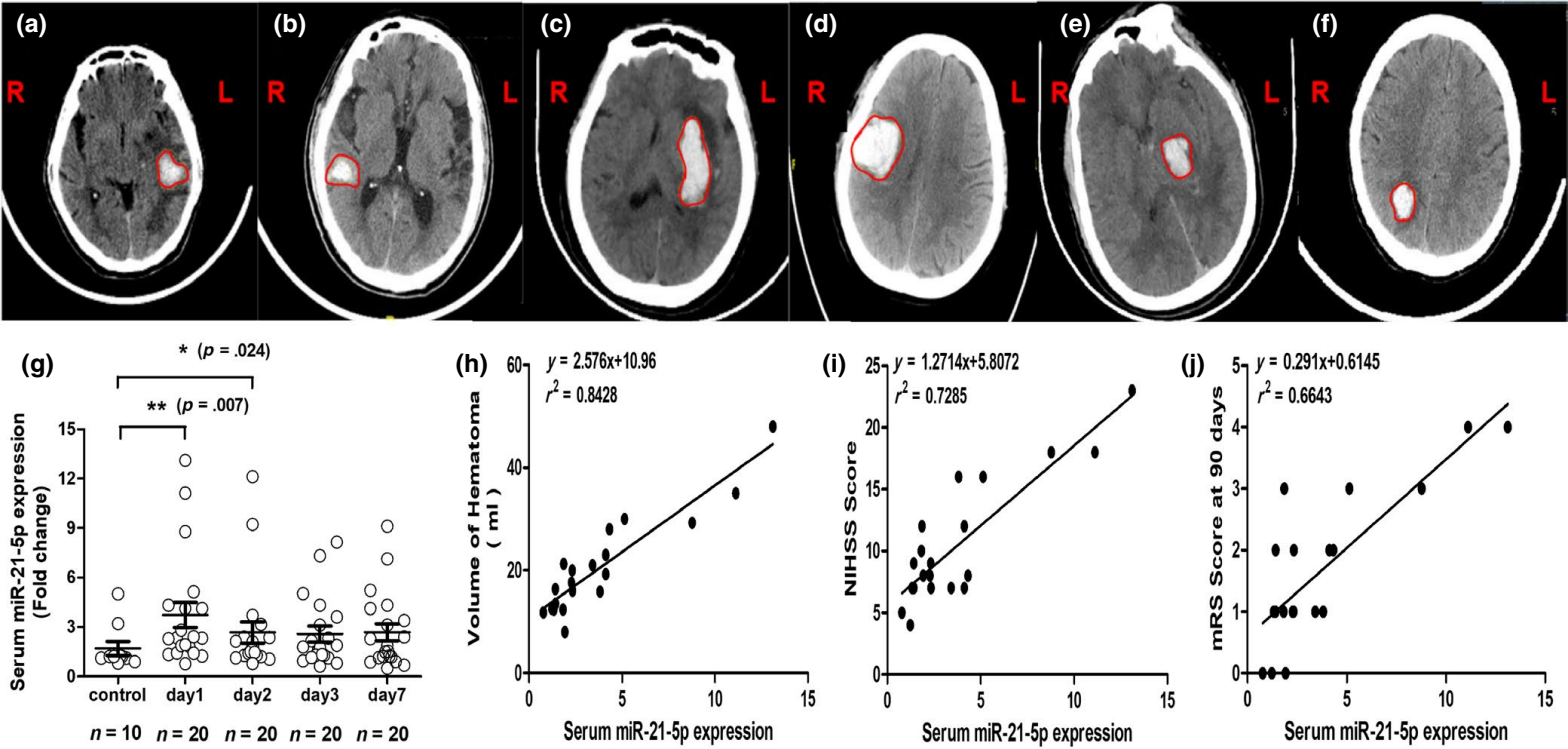
Serum miR‐21‐5p was elevated in elderly ICH patients and was associated with clinical outcomes. (a) Left frontal, temporal lobe cerebral haemorrhage, volume: 17.31 ml; (b) right temporal lobe haemorrhage, volume: 22.51 ml; (c) left basal ganglia cerebral haemorrhage, volume: 34.07 ml; (d) right frontal, parietal lobe cerebral haemorrhage, volume: 43.45 ml; (e) left thalamic cerebral haemorrhage, volume: 30.30 ml; (f) right parietal lobe cerebral haemorrhage, volume: 28.0 ml; (g) RT–PCR measured the serum miR‐21‐5p expression levels on days 1, 2, 3 and 7 from patients with cerebral haemorrhages (*n* = 20) and normal patients (*n* = 10); (h) serum miR‐21‐5p levels and haematoma volume were positively correlated in ICH patients; (i–j) Both NIHSS score and mRS score were correlated with serum miR‐21‐5p at 90 days in ICH patients

Next, we evaluated whether differential miR‐21‐5p expression is consistent with neurological deficit severity in ICH patients over 65 years old. Forty‐eight patients were evaluated, and twenty‐eight were excluded for lateral ventricular haemorrhage, brain stem haemorrhage, treatment with surgery or evacuation of the haematoma during the acute phase, or unavailable computed tomography (CT) results. Upon their first hospital admission, we collected the patients' imaging diagnoses and clinical data (Table S2 and Figure 5a–f). All CT results were obtained using the first admitting imaging diagnosis, and the median haematoma volume was 20.12 ± 12.34 ml.

MiR‐21‐5p levels were elevated in ICH patients, as confirmed by RT–qPCR (Figure 5g). Compared with healthy controls, miR‐21‐5p expression levels differed significantly on days 1 (*p* = 0.007), 2 (*p* = 0.024), 3 (*p* = 0.038) and 7 (*p* = 0.124) in patients with ICH. Overall, patients with ICH exhibited higher miR‐21‐5p expression levels than did healthy individuals during the acute phase, which peaked on the first day and then decreased slightly thereafter.

Spearman's correlation coefficient (*r* ^2^) was used to identify correlations between patient clinical outcomes and miR‐21‐5p expression levels in their serum. miR‐21‐5p levels were positively correlated with haematoma volume (*r* ^2^ = 0.8428; *p* < 0.001), the National Institutes of Health Stroke Scale (NIHSS) score (*r* ^2^ = 0.7285; *p* < 0.001) and the modified Rankin scale (mRS) score at 90 days (*r* ^2^ = 0.6643; *p* < 0.001; Figures 5h–j). MiR‐21‐5p remained correlated with the NIHSS scores (β = 0.742; *t* = 2.341; *p* < 0.05) and mRS scores (β = 0.412; *t* = 5.280; *p* < 0.05) in the multiple linear regression analysis after adjusting for baseline haematoma volume. These phenomena support the hypothesis that serum miR‐21‐5p levels are correlated with the neurological deficit severity (NIHSS scores) and clinical outcomes (mRS scores at 90 days) of elderly patients.

## 3 DISCUSSION

An increasing number of miRNAs have emerged as biomarkers for the diagnosis and prognosis of various neurological diseases (Wang et al., 2018; Xie et al., 2019). Accumulating data imply that differential miRNA expression profiles detected by sequencing are involved in developing cognitive defects (Tang et al., 2019) and Parkinson's disease (Su et al., 2018). In this study, we identified that miR‐21‐5p mediated haemin neuroprotection by targeting DUSP8 to alleviate neuroinflammation. Previous studies have reported that DUSP8 functions in various diseases. For example, in heart failure, DUSP8 repressed ventricular remodelling and disease susceptibility (Liu et al., 2016). DUSP8 also enhances protection and blocks ischaemia–reperfusion injury (Huang, Liu, Zhu, Wu, & Guo, 2013). In this study, we identified that miR‐21‐5p directly bound to and negatively regulated DUSP8 expression. In addition, miR‐21‐5p suppressed DUSP8 expression and negatively regulated MAPKs by dephosphorylating ERK1/2 signalling to activate the protective effect of HO‐1 signalling in haemorrhagic perifocal inflammation.

The potential neuroprotection from haemin treatment within the activated miR‐21‐5p‐DUSP8‐ERK1/2 signalling pathway after haemorrhagic damage is noteworthy. In contrast, with haemin treatment, miR‐21‐5p was knocked down by injecting A‐miR‐21‐5p into the lateral ventricles of rats before collagenase injection. A‐miR‐21‐5p effectively alleviated ICH‐induced injury in vivo. Both A‐miR‐21‐5p and haemin decreased brain haematoma volume and profoundly reduced neuronal death. In addition, A‐miR‐21‐5p preserved more BBB integrity than did haemin and reduced cerebral oedema after inducing ICH. Both A‐miR‐21‐5p and haemin therapy alleviated tissue iron deposition and accelerated haematoma absorption 10 days post‐ICH. Similarly, A‐miR‐21‐5p injection induced better neurological scores and motor coordination after ICH was induced in aged rats. Moreover, A‐miR‐21‐5p affected post‐ICH behavioural changes by greatly improving cognition, poststroke anxiety and memory deficit with aging. Consequently, both treatments contributed to protection from haemorrhagic damage and neurological deficits. In the water maze experiment, cognition and learning‐memory disorders were effectively reversed after haemin treatment, but the motor functions of the haemin group, such as movement velocity and distance, did not significantly improve. Surviving neurons likely caused unregulated haemoglobin synthesis in response to ICH‐associated stress, thus increasing the α‐globin levels in the diseased tissue. Ferritin upregulation in ICH‐senescent brains has been found to increase iron availability in the absence of globin‐chain upregulation and improve cognitive functions in aging (Huang Z et al., 2013).

The serum miR‐21‐5p response to inflammatory injury after a haemorrhage is an independent outcome predictor for aged patients, which is considered a new finding. The peak peripheral blood concentration time is related to the evolution of the brain's stress response, suggesting that miR‐21‐5p response and the stress response are interrelated, perhaps in the cases of BBB rupture, peripheral blood cells, astrocytes and glial cells, and is likely to be the miR‐21‐5p concentration source measured in the serum of aged patients. Thus, miR‐21‐5p was elevated in sera from elderly patients and was positively correlated with haematoma volume, neurological deficit severity (NIHSS scores) and clinical outcome (mRS scores at 90 days) compared with healthy individuals via Spearman's correlation analysis or multiple linear regression analysis. Early in the post‐ICH period, HO‐1 changes from a pro‐inflammatory phenotype to a phenotype that promotes wound healing and repair (Schallner et al., 2017). Thus, we hypothesized that an increase in miR‐21‐5p early in ICH activates HO‐1 to manifest this phenotype shift. In the advanced clinical prognoses of elderly patients, miR‐21‐5p levels may be lowered to modulate ICH‐induced inflammatory injury combined with haemin therapy.

Interestingly, while not all the functional verification experiments in the haemin group showed obvious differences, the BBB integrity (mean fluorescence intensity; MFI), neuronal death (TUNEL staining), haematoma absorption (Perls' Prussian blue staining) and cognitive memory disorder (swimming distances and crossing times in the water maze) were attenuated, which was consistent with our previous report (Yin, et al., 2015, 2017). However, miR‐21 inhibition treatment rescued cerebral oedema and neurological deficits (mNSS and corner test), implying that the effect of inducing A‐miR‐21‐5p is more specific for modulating HO‐1 neuroprotective functions. Because miRNAs have multiple targets (Urbich, Kuehbacher, & Dimmeler, 2008), we speculate that miR‐21‐5p acts directly on DUSP8 and affects other neuroprotective targets, such as TRPM7, ATF6 and NRF2. However, haemin treatment leads to changes in several miRNAs, which may generate antagonistic effects. Additionally, inhibiting miR‐21‐5p indirectly reversed ICH‐induced phosphorylation of ERK1/2 and augmented HO‐1 anti‐inflammatory pathway activation owing to DUSP8 repressing miR‐21‐5p, demonstrating a positive feedback loop. Modulating HO‐1 activity that is reversed by inhibiting miR‐21‐5p represents a molecular target for diminishing ICH‐induced oxidative and inflammatory injury.

HO‐1 activation induces miR‐21‐5p expression in cerebral haematomas to elicit anti‐inflammatory, antiapoptotic and antioxidant activity in haemin therapy, which DUSP8 modulates by suppressing phosphorylated molecules such as ERK1/2. Therefore, modulating HO‐1 activity via inhibiting miR‐21‐5p represents a molecular target for developing novel and more efficacious strategies to diminish ICH‐induced oxidative and inflammatory injury that has been aggravated by aging.

## 4 EXPERIMENTAL PROCEDURES

### 4.1 Materials and methods

#### 4.1.1 Study approval and animals

The Medical Ethics Committee of Nanchang University, Nanchang, China, approved all patient‐involved studies. The Animal Care and Use Committee of Nanchang University approved all laboratory animal procedures. Sixty 18‐month‐old male Sprague Dawley rats (weighing 500–600 g) were obtained from the Animal Center of Hubei Province.

#### 4.1.2 Patient blood sample collection

Elderly patients with acute cerebral haemorrhage were registered at the Second Affiliated Hospital of Nanchang University from September 2017 to March 2018.

The Medical Ethics Committee of the Second Affiliated Hospital of Nanchang University, Nanchang, China, approved all patient blood sample collections. Patients or their relatives signed written informed consent. Eligible patients were ≥65 years old and developed haemorrhage within 72 hr. Exclusion criteria were (a) subtentorial haematoma or brain ventricle haemorrhage; (b) cerebral haemorrhage due to arteriovenous malformation (AVM) or aneurysm and bleeding after cerebral infarction; (c) surgical intervention; (d) recurrent stroke within 3 months; or (e) refusal to participate. Patients were treated per the current ICH treatment guidelines. Functional outcomes included the NIHSS score on the first day after onset and the modified Rankin scale (mRS) and mortality on day 90. Three doctors who were blinded to the experimental design evaluated the CT images. Patient serum samples (*n* = 20) were obtained 1, 2, 3 and 7 days after admission, and healthy control samples (*n* = 10) were obtained 1 day after admission. The samples were centrifuged at 1,500 *r*/min for 10 min. Serum samples were obtained and preserved at −80°C.

#### 4.1.3 Lateral ventricles injected with A‐miR‐21‐5p and description of the animal model of ICH

A‐miR‐21‐5p (AntagomiR‐21‐5p, RiboBio) was injected into the lateral ventricles of rats to inhibit endogenous miR‐21‐5p in vivo. Rats were anaesthetized, and a stereotactic brain biopsy was performed. A 1‐mm hole was drilled with a dental drill using the coordinates of 1.6 mm posterior to the bregma, 1.1 mm right of the middle line and 4.5 mm deep from the skull surface. A‐miR‐21‐5p and the negative control (aNC) were dissolved in a final concentration of sterile saline 20 nmol/L. Five microlitres of either the mixture or saline was injected into the lateral ventricle at 0.5 µl/min. After 1 week, ICH was induced as previously described (Chen‐Roetling et al., 2017). In the right basal ganglia (stereotactic coordinates: 0.2 mm in the front of bregma, 3 mm right of the middle line, 6 mm deep), type VII collagenase (0.5 U in 2 ml of normal saline per rat) was infused into the brain at 0.4 µl/min. For the sham‐operated rats, a hole was drilled in the same position, and an equal amount of saline was injected into the brain.

The A‐miR‐21‐5p and miR‐NC sequences were Rno‐miR‐21‐5p antagomir (A‐miR‐21‐5p): UCAACAUCAGUCUGAUAAGCUA and antagomir negative control (miR‐NC): CAGUACUUUUGUGUAGUACAAA.

#### 4.1.4 Drug treatment

Haemin (Sigma‐Aldrich Chemical Co.) was titrated to pH 7.4 as a 2 mM solution in sodium phosphate buffer and further diluted to 20 mg/ml in sterile saline. Protoporphyrin IX zinc (II) (ZnPP; Sigma‐Aldrich) was diluted to 10 mg/ml in 35% dimethyl sulfoxide (DMSO). Within 3 hr of establishing the rat models, haemin or ZnPP was injected into the tail veins at the following doses: haemin, 26 mg/kg (per Lu, Chen‐Roetling, & Regan, 2014) and ZnPP, 10 mg/kg (per Zhang et al., 2016). For the DMSO control groups, 0.3 ml of DMSO was infused into the tail veins. After 24 hr, the rats were euthanized, and their brains were excised and prepared for RNA extraction.

### 4.2 Behavioural assessment

Behaviour was assessed 24 hr after inducing ICH using the modified Neurological Severity Score with the corner‐turning test to comprehensively evaluate motor, sensory, reflex and balance functions, as previously described. The percentage of right turns was then calculated for further analysis.

### 4.3 Injury volume assessment

Rats were euthanized 24 hr after ICH induction and miRNA treatment to assess the injury volume. The brains were sectioned 200 μm apart as previously described (Lu, Chen‐Roetling, & Regan, 2014). Sections were analysed using ImageJ software (version 1.50i; NIH, USA). The volume was measured by summing the areas multiplied by the interslice distance (200 μm).

### 4.4 Striatal water content

Rats were euthanized 24 hr after ICH induction, and the contralateral striata were excised and weighed. After drying in an oven at 95°C for 24 hr, the striatal tissues were reweighed using water content (%) = (wet weight‐dry weight)/wet weight × 100%.

### 4.5 Assessment of blood–brain barrier permeability

The Evans blue assay was performed per the method of Uyama et al. (1988). After inducing anaesthesia, the rats were immobilized on the operating table. Four millilitres per kilogram of 2% Evans blue was injected into the tail vein, turning the skin mucous membranes and limbs blue. Two hours later, the rats were perfused with 0.9% saline and 4% paraformaldehyde to remove the intravascular dye and to fix the tissues. Part of the tissue was homogenized in 200 μl of 50% trichloroacetic acid. After centrifugation, the supernatant was collected and diluted at 1:3 in 100% ethanol. Evans blue fluorescence (ex: 620 nm, em: 680 nm) was detected via spectrophotometer, data were analysed using Origin 7.0 software (OriginLab Co.), and MFI was calculated and normalized to the vehicle‐treated ICH controls. Another part of the brain tissue was cut into 10–20‐μm frozen slices and visualized using a laser scanning confocal microscope (550 nm, Olympus).

### 4.6 Prussian blue staining

Three similar frozen brain sections were collected per rat, and Prussian blue staining was performed per the manufacturer's instructions (Solarbio) and used to determine ferric iron deposition on day 10. Briefly, sections were rinsed three times in distilled water and incubated in Prussian blue solution (1% potassium ferrocyanide and 1% HCl [v/v]) for 20 min. Sections were then rinsed with distilled water three times and dehydrated through a series of ethanol gradients. Dimethylbenzene was used to transparentize the tissue, a neutral gum seal sheet was used, and the tissues were air‐dried. The dried samples were viewed microscopically, and images were collected for analysis.

### 4.7 TUNEL assay

The TUNEL assay was performed using the In Situ Cell Death Detection Kit (Roche) as per the manufacturer's instructions. In brief, the processed brains were paraffin‐embedded and sectioned into 5‐mm‐thick sections in phosphate‐buffered saline (PBS) and then immersed in TUNEL reaction mixture in the dark for 1 hr at 37°C. Labelled samples were viewed using a laser scanning confocal microscope and counted using ImageJ software.

### 4.8 HEK293T cell culture and cell transfection

HEK293T cells were cultured in Dulbecco's modified Eagle's medium (DMEM) (Invitrogen) supplemented with 1% glutamine (Invitrogen), 10% foetal bovine serum (FBS, Invitrogen), 100 U/ml penicillin (Invitrogen) and 100 μg/ml streptomycin (Invitrogen) at 37°C and 5% CO^2^. To determine the density, cells were transfected as per the instructions for the Lipofectamine 3000 (Invitrogen). Briefly, cells were seeded into 6‐well plates (4 × 10^5^/ml) and transfected with the plasmids (miR‐NC + DUSP8, miR‐21‐5p + DUSP8, miR‐21‐5p + Vector or miR‐NC + Vector) when the confluence reached 80%–90%. Transfection efficiency was measured 24 hr later. After 48 hr of transfection, we evaluated DUSP8 expression via qRT–PCR and Western blotting. All experiments were performed in triplicate.

The methods for the microarray analysis, differential miRNA candidate selection, bioinformatic analysis, immunofluorescence, ELISA, the Morris water maze, the luciferase reporter assay for miR‐21‐5p and DUSP8, RNA extraction, qRT–PCR, and the Western blot analysis are described in the Supplementary Materials.

### 4.9 Statistical analysis

SPSS 19.0 was used for statistical analysis. Univariate and multivariate linear regression were used to evaluate correlations between continuous variables. Two‐way analysis of variance (ANOVA) and a post hoc test were used to analyse the differences among groups. A *p*‐value of <0.05 was considered significant.

## AUTHORS’ CONTRIBUTIONS

Ling‐Qiang Zhu and Xiao‐ping Yin conceived and designed the work. Yetong Ouyang, Han Wang and Zhigang Wan conducted the experiments. Yetong Ouyang, Dongling Li, Yuqin Zhong, Min Yin and Qinghua Luo analysed and interpreted the data. Bing Bao, Zhiying Chen, Zhengyu Li and Zhengfang Qing advised on the experimental design and contributed materials and animals. Ling‐Qiang Zhu and Xiao‐ping Yin prepared the manuscript. All authors revised the manuscript and approved the final version.
