# Supplementary material for: MiR‐21‐5p/dual‐specificity phosphatase 8 signalling mediates the anti‐inflammatory effect of haem oxygenase‐1 in aged intracerebral haemorrhage rats
Source: Aging Cell. 2019 Aug 10;18(6):e13022. doi: 10.1111/acel.13022 (PMC6826124; doi:10.1111/acel.13022)
Supplement: Supplementary file 1 [file ACEL-18-e13022-s001.doc]

**Supplementary materials and methods**

**Microarray analysis and differential miRNA candidate selection**

Total RNA was extracted from haematoma tissues of the four groups (ICH, sham-surgery, and groups treated with hemin or ZnPP) using the miRcute miRNA Isolation Kit (TIANGEN, China). miRNA array analysis was conducted by CapitalBioCorp (Beijing, China). The raw intensity of each probe was processed using the R program (Version V3.4.2). Probes were normalized by 75% median scaling normalization criteria. Normalized spot intensities were transformed to miRNA expression log2 between each group. The spot ratios between the log2 ratio ≥1 or with log2ratio ≤-1 and P <0.05 were tested for additional analysis. The ICH-induced upregulated miRNAs (ICH vs sham) and hemin-induced downregulated miRNAs (hemin vs ICH) were identified. miRNAs (≥1.5-fold change) were selected for further qRT-PCR validation. CT values of the different samples were compared using the 2−ΔΔCT method with U6 snRNA expression levels used as an internal reference. Our experiments were performed in triplicate.

**Bioinformatic analysis**

The cluster Profiler R processing program was used to visualize the functional profiles (GO and KEGG) of target genes. The following criteria were used: more than two genes, false discovery rate (FDR) and P-values less than 0.05 and the target gene on the compiled data set of human interactome was used for PPI network construction and microarray data enrichment analysis. STRING v.10 was used to provide a critical evaluation and integration of protein-protein interactions online.

**Immunofluorescence**

Brain sections were post-fixed with 4% PFA and permeabilized with 0.3% Triton/PBS for 30 min at room temperature . Slides were then blocked in 2% normal goat serum for 2 hours at 37°C . Staining was performed with primary antibodies for Iba-1 (1:500, Abcam,ab5076) at 4°C overnight. Sections were then conjugated with their corresponding secondary antibody for fluorescent (Goat anti-Rabbit IgG–DyLight 488, 1:500, Invitrogen). Nuclei were stained with DAPI. Sections were examined under a fluorescence microscope.

**Enzyme-linked immunosorbent assay (ELISA)**

For IL-6,IL-1βand TNF-αmeasurement, the brains were in situ frozen and hematoma tissue samples were collected and homogenized. The amount of inflammatory cytokines were measured using Rat IL-6 ELISA kit (NeoBioscience, ERC003.48) ,Rat TNF-α ELISA kit(NeoBioscience, ERC102a.48)and Rat IL-1β ELISA kit (NeoBioscience, ERC007.48) respectively, according to the manufacturer’s instructions.

**Morris water maze**

In the hidden platform training, if the rat failed to find a submerged platform within 90 s, the rat would be guided to the platform and allowed to stand on it for 30 s. For 6 days after invisible training, the rat would rest for 1 day, and on day 8, a probe test was conducted. The platform was removed, and the rat was allowed to search for the platform for 90 s. The water maze image automatic collection and analysis system was provided by Xinsoft Technology Co., Ltd. (Shanghai, China)

**Luciferase reporter assay for miR-21-5p and DUSP8**

Before the luciferase reporter assay, we constructed a luciferase vector that included wild-type DUSP8 (pmirGLO-DUSP8-WT) and mutant-type DUSP8 (pmirGLO-DUSP8-MUT). miR-21-5p mimics, inhibitors and the negative control were analysed using RiboBio (Guangzhou, China). The miR-21-5p mimics and the indicated wild-type or mutated 3′ UTR luciferase vector were co-transfected into HEK293T cells with renilla luciferase (pRLTK Vector, Promega, USA) as a transfection efficiency control. After 24 h of transfection, cells were harvested to determine the luciferase activity according to the Dual Luciferase Reporter Assay System (Promega, WI, USA). Each transfection was performed in triplicate.

**RNA extraction and qRT-PCR**

The extraction of total RNA from serum, rat brain tissue, and HEK293T was performed using the miRcute miRNA Isolation Kit (TIANGEN, Lot:DP501, Beijing, China) according to the manufacturer’s instructions. Total RNA (2 μg) was reverse transcribed for miRNA using a miRcute Enhanced miRNA First-Strand cDNA Synthesis Kit (TIANGEN, Lot: KR211, Beijing, China). Total RNA (10 μg) was reverse transcribed for mRNA using the QuantiNova™ Reverse Transcription Kit (QIAGEN, ID: 205410, Germany). The SYBR Green I fluorescent dye method (SYBR® PrimeScript™ RT-PCR Kit (Takara Biotechnology CO.) was used for PCR detection. Amplification of miRNA/mRNA was performed on an Applied Biosystems StepOne™ Real-Time PCR System (Applied Biosystems). The following cycling conditions were 95°C for 2 min, followed by 40 cycles of 95°C for 15 s, 60°C for 20 s and 72°C for 20 s for miRNA and 95°C for 2 min, followed by 40 cycles of 95°C for 15 s, TM for 30 s and 72°C for 25 s for mRNA. U6 snRNA/GAPDH served as the internal control for RNA quantification. The relative concentration was calculated by the 2-∆∆CT method.

**Western blot analysis**

First, brain tissue and HEK293T were lysed in lysis buffer. The protein concentration was determined using the Bio-Rad Protein Assay Kit according to the manufacturer’s protocol; 20 μg of protein from each sample was separated via 10%-15% SDS-PAGE and then transferred to a nitrocellulose membrane. The membranes were blocked in TBST with 5% BSA for 1 h and then incubated with primary antibodies against β-actin (Abcam, 1:200), HO-1 (Abcam, 1:200), DUSP8 (Novus, 1:200), ERK (Abcam, 1:200), and p-ERK (Abcam, 1:200) overnight at 4°C in TBST. After three washes with TBST, membranes were probed with horseradish peroxidase-conjugated anti-rabbit (1:1000; Sigma) or anti-mouse (1:1000; Sigma) secondary antibody for 1 h at room temperature. Immunoreactive bands were visualized using the Odyssey Infrared Imaging System (Licor Biosciences, Lincoln, NE).

**
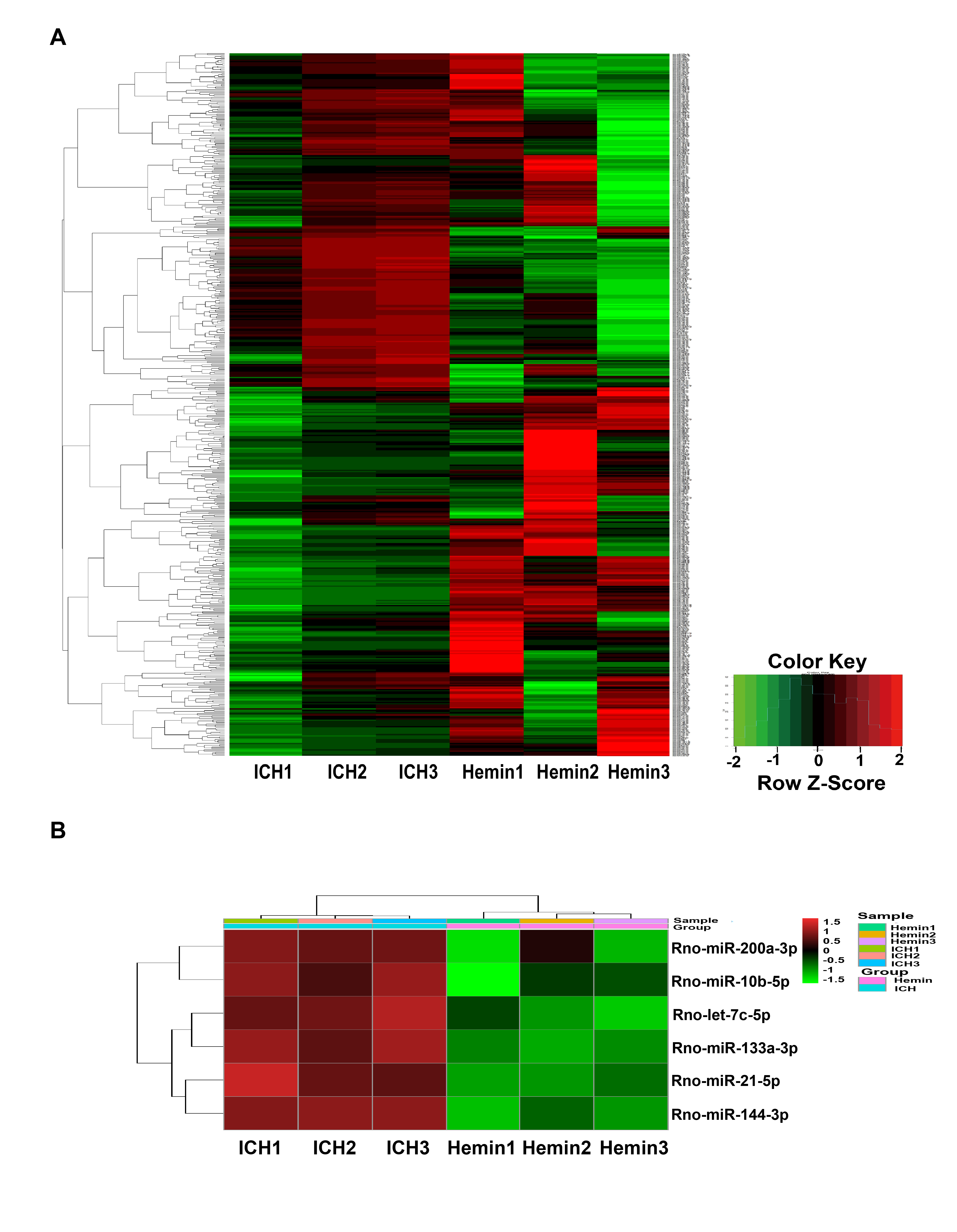
Supplementary Fig1.The expression profiles of miRNAs upon HO-1 activation.**

Heatmap visualization of miRNA expression pattern between the ICH-induced upregulated miRNAs (ICH vs sham) and hemin-induced downregulated miRNAs (hemin vs ICH) analyzed with R software.For heatmaps, columns denote samples and rows represent individual miRNAs; Color key indicates gene expression values with green: lowest and red: highest.

**

**

**Supplementary Fig2.Gene-enrichment analysis result of miR-21-5p-regulated biomolecular network.**

1. Top10 statistically enriched KEGG pathways.
2. Top10 statistically enriched biological processes, molecular functions and cellular component categorization. (The top ten items were selected to analyse and visualize functional profiles of genes according to the P-value < 0.05.)
3. Protein-protein interaction network of 20 gene clusters that are markedly related to the nervous system by web-STRING. Network nodes represent proteins (shown in gene names in the figure), edges represent protein–protein associations. Edges in different colors represent associations identified by different methods.


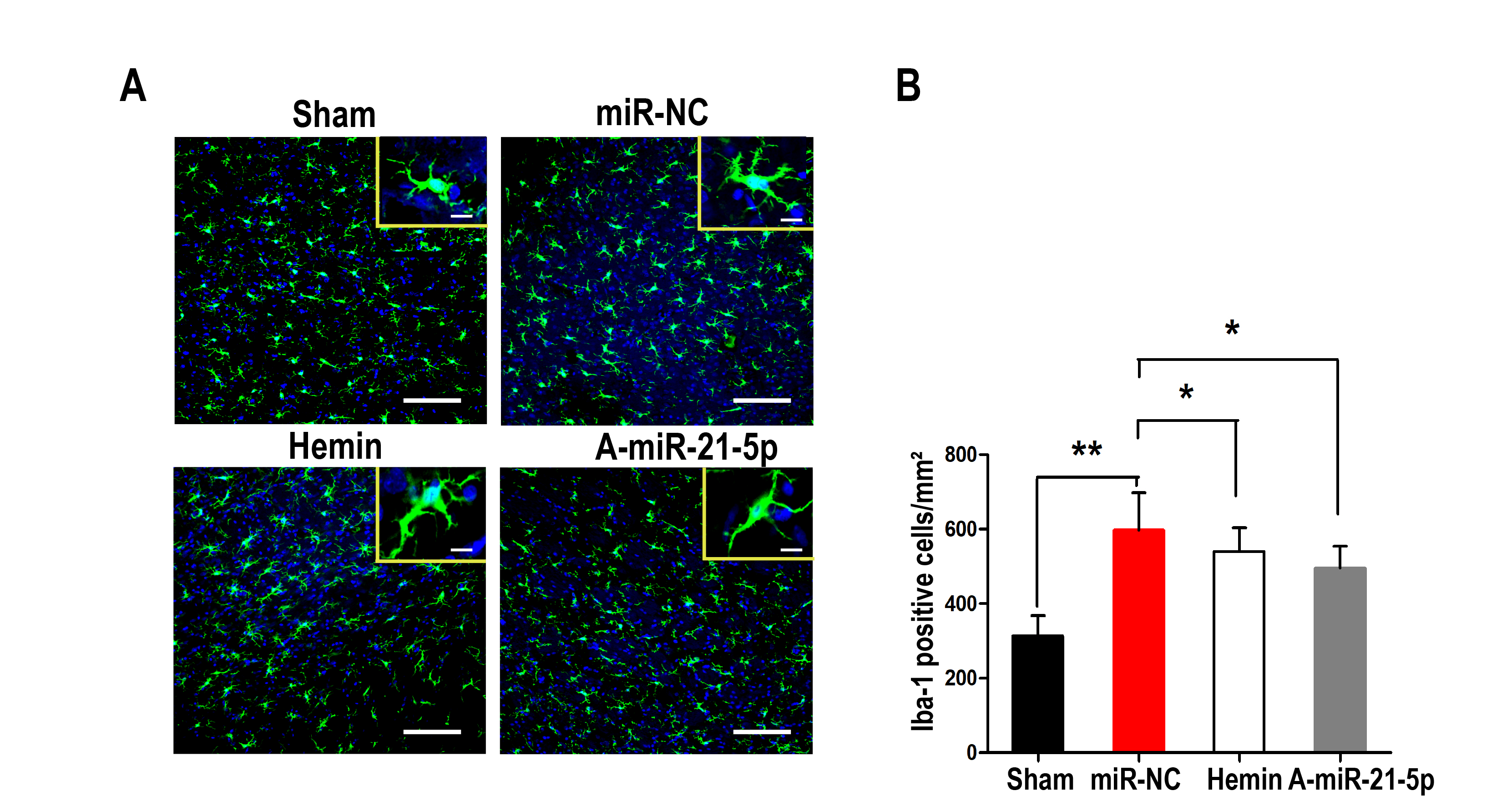


**Supplementary Fig3. The extent of neuroinflammation depends on different**

**treatments.**

Immunofluorescence staining for Iba-1 (green) showing the morphology and quantity of microglial cells post-ICH in the perihematoma region at the treatment of the Sham group , miR-NC, Hemin,and A-miR-21-5p. Nuclei were stained with DAPI (blue). Higher magnification images show the representative changes in microglial morphology. All images are representative of 3 to 4 rats per group with 3 sections per brain. Bar graph shows the number of Iba1+ cells in four groups. ** indicates P < 0.01. * indicates P < 0.05. Scale bars, 50μm .

**
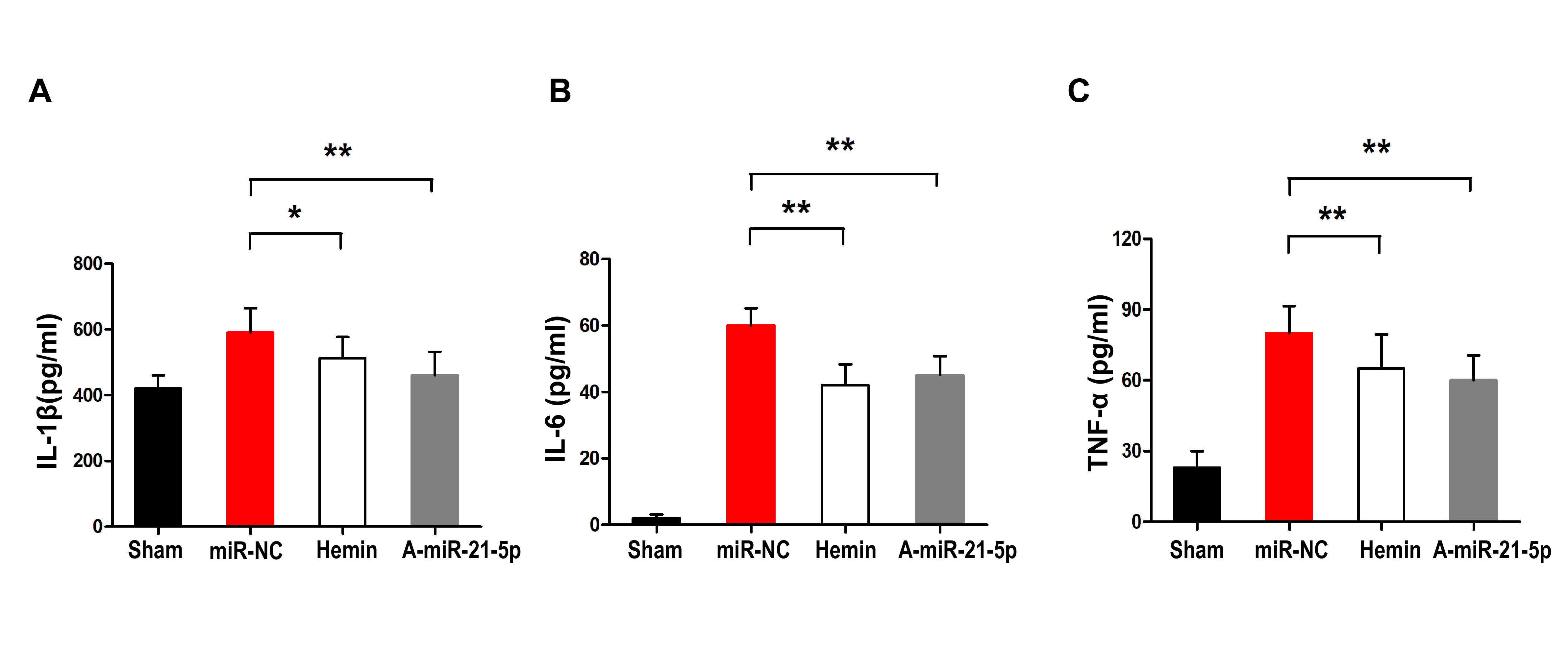
**

**Supplementary Fig4. Decreased inflammation factor IL-1β, IL-6, and TNF-α activity following treatment.**ELISA assay for IL-1β (A), IL-6(B) and TNF-α(C) activity levels in the perihematoma region in sham, miR-NC, Hemin, A-miR-21-5p rats group 24 hours post-ICH, *P< 0.05,**P < 0.01, n = 3 per group; data represented as mean ± standard error)

**Supplementary Table1. List of predicted target genes of miR-21-5p from the TargetScan\PITA\ Miranda databases.**

| **Target gene** | **Representative transcript** | **Gene name** | **Total context++ score** | **Aggregate PCT** |
| --- | --- | --- | --- | --- |
| YOD1 | ENST00000315927.4 | YOD1 deubiquitinase | -0.72 | 0.94 |
| DUSP8 | ENST00000397374.3 | dual specificity phosphatase 8 | -0.55 | 0.81 |
| SCML2 | ENST00000398048.3 | sex comb on midleg-like 2 (Drosophila) | -0.64 | 0.81 |
| CCL1 | ENST00000225842.3 | chemokine (C-C motif) ligand 1 | -0.59 | 0.74 |
| IL12A | ENST00000466512.1 | interleukin 12A (natural killer cell stimulatory factor 1, cytotoxic lymphocyte maturation factor 1, p35) | -0.61 | 0.74 |
| FASLG | ENST00000340030.3 | Fas ligand (TNF superfamily, member 6) | -0.57 | 0.51 |
| GPR64 | ENST00000379873.2 | G protein-coupled receptor 64 | -0.88 | 0.46 |
| ARMCX1 | ENST00000372829.3 | armadillo repeat containing, X-linked 1 | -0.68 | 0.3 |
| AGO2 | ENST00000220592.5 | argonaute RISC catalytic component 2 | -0.56 | 0.24 |
| AIM1L | ENST00000527815.1 | absent in melanoma 1-like | -0.55 | < 0.1 |
| MPRIP | ENST00000341712.4 | myosin phosphatase Rho interacting protein | -0.21 | 0.85 |
| TGFBI | ENST00000442011.2 | transforming growth factor, beta-induced, 68kDa | -0.5 | 0.83 |
| ARHGAP24 | ENST00000395184.1 | Rho GTPase activating protein 24 | -0.44 | 0.83 |
| ELF2 | ENST00000394235.2 | E74-like factor 2 (ets domain transcription factor) | -0.43 | 0.83 |
| SKI | ENST00000378536.4 | v-ski avian sarcoma viral oncogene homolog | -0.38 | 0.83 |
| CSRNP3 | ENST00000314499.7 | cysteine-serine-rich nuclear protein 3 | -0.16 | 0.83 |
| SMAD7 | ENST00000262158.2 | SMAD family member 7 | -0.52 | 0.8 |
| SLC30A10 | ENST00000366926.3 | solute carrier family 30, member 10 | -0.35 | 0.8 |
| JHDM1D | ENST00000397560.2 | jumonji C domain containing histone demethylase 1 homolog D (S. cerevisiae) | -0.33 | 0.8 |
| GABRB2 | ENST00000393959.1 | gamma-aminobutyric acid (GABA) A receptor, beta 2 | -0.19 | 0.8 |
| IL6R | ENST00000344086.4 | interleukin 6 receptor | -0.29 | 0.78 |
| THRB | ENST00000396671.2 | thyroid hormone receptor, beta | -0.4 | 0.77 |
| GATAD2B | ENST00000368655.4 | GATA zinc finger domain containing 2B | -0.39 | 0.77 |
| RALGPS2 | ENST00000367635.3 | Ral GEF with PH domain and SH3 binding motif 2 | -0.38 | 0.77 |
| RASGRP1 | ENST00000559830.1 | RAS guanyl releasing protein 1 (calcium and DAG-regulated) | -0.25 | 0.77 |
| FMN1 | ENST00000334528.9 | formin 1 | -0.19 | 0.77 |
| CASKIN1 | ENST00000343516.6 | CASK interacting protein 1 | -0.28 | 0.76 |
| NFIB | ENST00000397575.3 | nuclear factor I/B | -0.39 | 0.75 |
| SOX5 | ENST00000546136.1 | SRY (sex determining region Y)-box 5 | -0.35 | 0.74 |
| STRN | ENST00000263918.4 | striatin, calmodulin binding protein | -0.03 | 0.74 |
| PELI1 | ENST00000358912.4 | pellino E3 ubiquitin protein ligase 1 | -0.37 | 0.73 |
| PURB | ENST00000395699.2 | purine-rich element binding protein B | -0.13 | 0.73 |
| ZNF367 | ENST00000375256.4 | zinc finger protein 367 | -0.54 | 0.71 |
| KLHL15 | ENST00000328046.8 | kelch-like family member 15 | -0.42 | 0.71 |
| STAG2 | ENST00000371160.1 | stromal antigen 2 | -0.37 | 0.71 |
| MAP3K1 | ENST00000399503.3 | mitogen-activated protein kinase kinase kinase 1, E3 ubiquitin protein ligase | -0.29 | 0.71 |
| JAG1 | ENST00000254958.5 | jagged 1 | -0.27 | 0.71 |
| SPRY1 | ENST00000394339.2 | sprouty homolog 1, antagonist of FGF signaling (Drosophila) | -0.51 | 0.7 |
| NFIA | ENST00000403491.3 | nuclear factor I/A | -0.24 | 0.7 |
| VCL | ENST00000372755.3 | vinculin | -0.52 | 0.69 |
| SPRY2 | ENST00000377102.1 | sprouty homolog 2 (Drosophila) | -0.35 | 0.69 |
| EHD1 | ENST00000320631.3 | EH-domain containing 1 | -0.27 | 0.69 |
| KBTBD6 | ENST00000379485.1 | kelch repeat and BTB (POZ) domain containing 6 | -0.29 | 0.64 |
| RECK | ENST00000377966.3 | reversion-inducing-cysteine-rich protein with kazal motifs | -0.42 | 0.63 |
| TMEM170A | ENST00000357613.4 | transmembrane protein 170A | -0.5 | 0.62 |
| PLAG1 | ENST00000316981.3 | pleiomorphic adenoma gene 1 | -0.46 | 0.62 |
| CDC25A | ENST00000302506.3 | cell division cycle 25A | -0.21 | 0.62 |
| ADNP | ENST00000371602.4 | activity-dependent neuroprotector homeobox | -0.31 | 0.61 |
| SGMS2 | ENST00000394684.4 | sphingomyelin synthase 2 | -0.29 | 0.61 |
| MAP2K3 | ENST00000342679.4 | mitogen-activated protein kinase kinase 3 | -0.33 | 0.58 |
| NPAS3 | ENST00000346562.2 | neuronal PAS domain protein 3 | -0.14 | 0.57 |
| PLEKHA1 | ENST00000538022.1 | pleckstrin homology domain containing, family A (phosphoinositide binding specific) member 1 | -0.36 | 0.55 |
| KBTBD7 | ENST00000379483.3 | kelch repeat and BTB (POZ) domain containing 7 | -0.34 | 0.55 |
| PCSK6 | ENST00000348070.1 | proprotein convertase subtilisin/kexin type 6 | -0.3 | 0.55 |
| PAG1 | ENST00000220597.4 | phosphoprotein associated with glycosphingolipid microdomains 1 | -0.17 | 0.55 |
| MTMR12 | ENST00000280285.5 | myotubularin related protein 12 | -0.12 | 0.54 |
| PIK3R1 | ENST00000521381.1 | phosphoinositide-3-kinase, regulatory subunit 1 (alpha) | -0.07 | 0.54 |
| BCL7A | ENST00000538010.1 | B-cell CLL/lymphoma 7A | -0.27 | 0.53 |
| KRIT1 | ENST00000394507.1 | KRIT1, ankyrin repeat containing | -0.28 | 0.52 |
| SATB1 | ENST00000338745.6 | SATB homeobox 1 | -0.27 | 0.52 |
| OSR1 | ENST00000272223.2 | odd-skipped related 1 (Drosophila) | -0.26 | 0.52 |
| ALX1 | ENST00000316824.3 | ALX homeobox 1 | -0.24 | 0.52 |
| TERF2 | ENST00000254942.3 | telomeric repeat binding factor 2 | -0.23 | 0.52 |
| ARHGEF12 | ENST00000397843.2 | Rho guanine nucleotide exchange factor (GEF) 12 | -0.23 | 0.52 |
| ZFP36L2 | ENST00000282388.3 | ZFP36 ring finger protein-like 2 | -0.22 | 0.52 |
| TAGAP | ENST00000367066.3 | T-cell activation RhoGTPase activating protein | -0.22 | 0.52 |
| CHD7 | ENST00000423902.2 | chromodomain helicase DNA binding protein 7 | -0.22 | 0.52 |
| SNX29 | ENST00000566228.1 | sorting nexin 29 | -0.19 | 0.52 |
| SESN3 | ENST00000536441.1 | sestrin 3 | -0.18 | 0.52 |
| TIAM1 | ENST00000286827.3 | T-cell lymphoma invasion and metastasis 1 | -0.17 | 0.52 |
| C20orf194 | ENST00000453730.2 | chromosome 20 open reading frame 194 | -0.16 | 0.52 |
| CADM1 | ENST00000452722.3 | cell adhesion molecule 1 | -0.16 | 0.52 |
| TNKS | ENST00000310430.6 | tankyrase, TRF1-interacting ankyrin-related ADP-ribose polymerase | -0.15 | 0.52 |
| FTO | ENST00000471389.1 | fat mass and obesity associated | -0.15 | 0.52 |
| WNT2B | ENST00000369686.5 | wingless-type MMTV integration site family, member 2B | -0.13 | 0.52 |
| DNAJA2 | ENST00000317089.5 | DnaJ (Hsp40) homolog, subfamily A, member 2 | -0.13 | 0.52 |
| TRPM7 | ENST00000560955.1 | transient receptor potential cation channel, subfamily M, member 7 | -0.12 | 0.52 |
| TNRC6B | ENST00000335727.9 | trinucleotide repeat containing 6B | -0.11 | 0.52 |
| STK40 | ENST00000359297.2 | serine/threonine kinase 40 | -0.1 | 0.52 |
| TNPO1 | ENST00000337273.5 | transportin 1 | -0.1 | 0.52 |
| TGFBR2 | ENST00000359013.4 | transforming growth factor, beta receptor II (70/80kDa) | -0.05 | 0.52 |
| DNAJC16 | ENST00000375847.3 | DnaJ (Hsp40) homolog, subfamily C, member 16 | -0.05 | 0.52 |
| UBE2D3 | ENST00000453744.2 | ubiquitin-conjugating enzyme E2D 3 | -0.52 | 0.51 |
| PVRL3 | ENST00000319792.3 | poliovirus receptor-related 3 | -0.39 | 0.51 |
| TRAPPC8 | ENST00000283351.4 | trafficking protein particle complex 8 | -0.37 | 0.51 |
| HNRNPK | ENST00000376281.4 | heterogeneous nuclear ribonucleoprotein K | -0.31 | 0.51 |
| KLF6 | ENST00000542957.1 | Kruppel-like factor 6 | -0.26 | 0.51 |
| KLHL42 | ENST00000381271.2 | kelch-like family member 42 | -0.26 | 0.51 |
| MEF2C | ENST00000340208.5 | myocyte enhancer factor 2C | -0.24 | 0.51 |
| FBXO11 | ENST00000402508.1 | F-box protein 11 | -0.23 | 0.51 |
| RBPJ | ENST00000504907.1 | recombination signal binding protein for immunoglobulin kappa J region | -0.22 | 0.51 |
| MBNL1 | ENST00000357472.3 | muscleblind-like splicing regulator 1 | -0.21 | 0.51 |
| WWP1 | ENST00000517970.1 | WW domain containing E3 ubiquitin protein ligase 1 | -0.2 | 0.51 |
| RPS6KA3 | ENST00000379565.3 | ribosomal protein S6 kinase, 90kDa, polypeptide 3 | -0.1 | 0.51 |
| NTF3 | ENST00000423158.3 | neurotrophin 3 | -0.47 | 0.5 |
| PITX2 | ENST00000394595.3 | paired-like homeodomain 2 | -0.36 | 0.5 |
| CHIC1 | ENST00000373504.6 | cysteine-rich hydrophobic domain 1 | -0.31 | 0.5 |
| XKR6 | ENST00000304437.2 | XK, Kell blood group complex subunit-related family, member 6 | -0.27 | 0.5 |
| C1orf112 | ENST00000359326.4 | chromosome 1 open reading frame 112 | -0.25 | 0.5 |
| PAIP2B | ENST00000244221.8 | poly(A) binding protein interacting protein 2B | -0.22 | 0.5 |
| LCOR | ENST00000371103.3 | ligand dependent nuclear receptor corepressor | -0.19 | 0.5 |
| PPP3CA | ENST00000512215.1 | protein phosphatase 3, catalytic subunit, alpha isozyme | -0.16 | 0.5 |
| ROR1 | ENST00000371079.1 | receptor tyrosine kinase-like orphan receptor 1 | -0.11 | 0.5 |
| RNF20 | ENST00000389120.3 | ring finger protein 20, E3 ubiquitin protein ligase | -0.47 | 0.49 |
| PBRM1 | ENST00000356770.4 | polybromo 1 | -0.32 | 0.49 |
| ZCCHC3 | ENST00000382352.3 | zinc finger, CCHC domain containing 3 | -0.23 | 0.49 |
| GATA2 | ENST00000341105.2 | GATA binding protein 2 | -0.19 | 0.49 |
| KCNK10 | ENST00000340700.5 | potassium channel, subfamily K, member 10 | -0.11 | 0.49 |
| SPPL3 | ENST00000353487.2 | signal peptide peptidase like 3 | -0.05 | 0.49 |
| ZKSCAN5 | ENST00000394170.2 | zinc finger with KRAB and SCAN domains 5 | -0.34 | 0.48 |
| JPH1 | ENST00000342232.4 | junctophilin 1 | -0.27 | 0.48 |
| ZNF704 | ENST00000327835.3 | zinc finger protein 704 | -0.17 | 0.48 |
| SLC10A7 | ENST00000264986.3 | solute carrier family 10, member 7 | -0.3 | 0.47 |
| NAA50 | ENST00000240922.3 | N(alpha)-acetyltransferase 50, NatE catalytic subunit | -0.26 | 0.47 |
| RPP25 | ENST00000322177.5 | ribonuclease P/MRP 25kDa subunit | -0.11 | 0.47 |
| RAB11A | ENST00000569896.1 | RAB11A, member RAS oncogene family | -0.39 | 0.45 |
| SRSF3 | ENST00000373715.6 | serine/arginine-rich splicing factor 3 | -0.42 | 0.44 |
| PJA2 | ENST00000361189.2 | praja ring finger 2, E3 ubiquitin protein ligase | -0.26 | 0.43 |
| CNTFR | ENST00000351266.4 | ciliary neurotrophic factor receptor | -0.28 | 0.42 |
| PHF14 | ENST00000403050.3 | PHD finger protein 14 | -0.16 | 0.42 |
| PCBP2 | ENST00000455667.3 | poly(rC) binding protein 2 | -0.4 | 0.4 |
| CRLS1 | ENST00000378863.4 | cardiolipin synthase 1 | -0.27 | 0.4 |
| CBX4 | ENST00000269397.4 | chromobox homolog 4 | -0.26 | 0.4 |
| MTAP | ENST00000380172.4 | methylthioadenosine phosphorylase | -0.25 | 0.4 |
| DAG1 | ENST00000515359.2 | dystroglycan 1 (dystrophin-associated glycoprotein 1) | -0.07 | 0.4 |
| SUZ12 | ENST00000322652.5 | SUZ12 polycomb repressive complex 2 subunit | -0.19 | 0.39 |
| KCNA1 | ENST00000382545.3 | potassium voltage-gated channel, shaker-related subfamily, member 1 (episodic ataxia with myokymia) | -0.15 | 0.38 |
| CNOT6 | ENST00000393356.1 | CCR4-NOT transcription complex, subunit 6 | -0.09 | 0.38 |
| RTF1 | ENST00000389629.4 | Rtf1, Paf1/RNA polymerase II complex component, homolog (S. cerevisiae) | -0.17 | 0.37 |
| STAT3 | ENST00000585517.1 | signal transducer and activator of transcription 3 (acute-phase response factor) | -0.14 | 0.36 |
| RNF111 | ENST00000348370.4 | ring finger protein 111 | -0.09 | 0.35 |
| HRK | ENST00000257572.5 | harakiri, BCL2 interacting protein (contains only BH3 domain) | -0.32 | 0.34 |
| RASA1 | ENST00000456692.2 | RAS p21 protein activator (GTPase activating protein) 1 | -0.14 | 0.34 |
| MYO5A | ENST00000399231.3 | myosin VA (heavy chain 12, myoxin) | -0.12 | 0.34 |
| HNRNPU | ENST00000444376.2 | heterogeneous nuclear ribonucleoprotein U (scaffold attachment factor A) | -0.12 | 0.34 |
| SRL | ENST00000399609.3 | sarcalumenin | -0.4 | 0.33 |
| LRRC57 | ENST00000397130.3 | leucine rich repeat containing 57 | -0.39 | 0.33 |
| RNF24 | ENST00000336095.6 | ring finger protein 24 | -0.39 | 0.33 |
| GABPB2 | ENST00000368918.3 | GA binding protein transcription factor, beta subunit 2 | -0.04 | 0.33 |
| FAM63B | ENST00000559228.1 | family with sequence similarity 63, member B | -0.15 | 0.32 |
| ZADH2 | ENST00000322342.3 | zinc binding alcohol dehydrogenase domain containing 2 | -0.11 | 0.32 |
| HS3ST1 | ENST00000002596.5 | heparan sulfate (glucosamine) 3-O-sulfotransferase 1 | -0.18 | 0.31 |
| AKIRIN1 | ENST00000432648.3 | akirin 1 | -0.14 | 0.31 |
| PPP1R3A | ENST00000284601.3 | protein phosphatase 1, regulatory subunit 3A | -0.28 | 0.3 |
| SOX7 | ENST00000554914.1 | Transcription factor SOX-7; Uncharacterized protein; cDNA FLJ58508, highly similar to Transcription factor SOX-7 | -0.26 | 0.3 |
| POM121C | ENST00000257665.5 | POM121 transmembrane nucleoporin C | -0.11 | 0.3 |
| AP3M1 | ENST00000355264.4 | adaptor-related protein complex 3, mu 1 subunit | -0.27 | 0.29 |
| MSX1 | ENST00000382723.4 | msh homeobox 1 | -0.24 | 0.29 |
| PSPC1 | ENST00000338910.4 | paraspeckle component 1 | -0.24 | 0.27 |
| CREBRF | ENST00000540014.1 | CREB3 regulatory factor | -0.17 | 0.27 |
| BCL2 | ENST00000398117.1 | B-cell CLL/lymphoma 2 | -0.14 | 0.27 |
| FZD8 | ENST00000374694.1 | frizzled family receptor 8 | -0.05 | 0.27 |
| TNS1 | ENST00000171887.4 | tensin 1 | -0.03 | 0.27 |
| FRS2 | ENST00000550389.1 | fibroblast growth factor receptor substrate 2 | -0.19 | 0.26 |
| SERP1 | ENST00000239944.2 | stress-associated endoplasmic reticulum protein 1 | -0.18 | 0.26 |
| KIAA1468 | ENST00000398130.2 | KIAA1468 | -0.12 | 0.26 |
| IFFO2 | ENST00000455833.2 | intermediate filament family orphan 2 | -0.07 | 0.26 |
| GPR125 | ENST00000334304.5 | G protein-coupled receptor 125 | -0.22 | 0.25 |
| SOX2 | ENST00000325404.1 | SRY (sex determining region Y)-box 2 | -0.21 | 0.25 |
| FAM107B | ENST00000378470.1 | family with sequence similarity 107, member B | -0.05 | 0.25 |
| UNKL | ENST00000389221.4 | unkempt homolog (Drosophila)-like | -0.24 | 0.24 |
| OLR1 | ENST00000543993.1 | oxidized low density lipoprotein (lectin-like) receptor 1 | -0.19 | 0.24 |
| PDZD8 | ENST00000334464.5 | PDZ domain containing 8 | -0.13 | 0.24 |
| SMARCD1 | ENST00000394963.4 | SWI/SNF related, matrix associated, actin dependent regulator of chromatin, subfamily d, member 1 | -0.11 | 0.24 |
| MSL1 | ENST00000398532.4 | male-specific lethal 1 homolog (Drosophila) | -0.1 | 0.24 |
| MAPK1 | ENST00000215832.6 | mitogen-activated protein kinase 1 | -0.02 | 0.24 |
| NKX6-1 | ENST00000515820.2 | NK6 homeobox 1 | -0.2 | 0.23 |
| CXCL10 | ENST00000306602.1 | chemokine (C-X-C motif) ligand 10 | -0.2 | 0.23 |
| RBMS3 | ENST00000396583.3 | RNA binding motif, single stranded interacting protein 3 | -0.2 | 0.23 |
| PURA | ENST00000331327.3 | purine-rich element binding protein A | -0.18 | 0.23 |
| C16orf52 | ENST00000542527.2 | chromosome 16 open reading frame 52 | -0.14 | 0.23 |
| BAHD1 | ENST00000416165.1 | bromo adjacent homology domain containing 1 | -0.12 | 0.23 |
| CD47 | ENST00000361309.5 | CD47 molecule | -0.12 | 0.23 |
| NBEA | ENST00000379939.2 | neurobeachin | -0.11 | 0.23 |
| THBD | ENST00000377103.2 | thrombomodulin | -0.09 | 0.23 |
| PRPF4B | ENST00000337659.6 | pre-mRNA processing factor 4B | -0.03 | 0.23 |
| BCL11B | ENST00000357195.3 | B-cell CLL/lymphoma 11B (zinc finger protein) | -0.02 | 0.23 |
| CPEB3 | ENST00000412050.4 | cytoplasmic polyadenylation element binding protein 3 | -0.27 | 0.22 |
| CD97 | ENST00000357355.3 | CD97 molecule | -0.2 | 0.22 |
| GLYR1 | ENST00000436648.5 | glyoxylate reductase 1 homolog (Arabidopsis) | -0.16 | 0.22 |
| C11orf87 | ENST00000327419.6 | chromosome 11 open reading frame 87 | -0.14 | 0.22 |
| TRIM33 | ENST00000358465.2 | tripartite motif containing 33 | -0.12 | 0.22 |
| CADM2 | ENST00000383699.3 | cell adhesion molecule 2 | -0.1 | 0.22 |
| ROBO2 | ENST00000461745.1 | roundabout, axon guidance receptor, homolog 2 (Drosophila) | -0.09 | 0.22 |
| SEMA3A | ENST00000265362.4 | sema domain, immunoglobulin domain (Ig), short basic domain, secreted, (semaphorin) 3A | -0.03 | 0.22 |
| MIA3 | ENST00000344922.5 | melanoma inhibitory activity family, member 3 | -0.32 | 0.21 |
| EGR3 | ENST00000519492.1 | early growth response 3 | -0.21 | 0.21 |
| ABCD2 | ENST00000308666.3 | ATP-binding cassette, sub-family D (ALD), member 2 | -0.14 | 0.21 |
| PDZD2 | ENST00000438447.1 | PDZ domain containing 2 | -0.03 | 0.21 |
| BNC2 | ENST00000380672.4 | basonuclin 2 | -0.06 | 0.18 |
| HGF | ENST00000222390.5 | hepatocyte growth factor (hepapoietin A; scatter factor) | -0.02 | 0.18 |
| SPG20 | ENST00000438666.2 | spastic paraplegia 20 (Troyer syndrome) | -0.38 | 0.16 |
| BOLL | ENST00000392296.4 | bol, boule-like (Drosophila) | -0.11 | 0.14 |
| MYT1L | ENST00000399161.2 | myelin transcription factor 1-like | -0.1 | 0.13 |
| FOXG1 | ENST00000382535.3 | forkhead box G1 | -0.02 | 0.13 |
| SOWAHC | ENST00000356454.3 | sosondowah ankyrin repeat domain family member C | -0.22 | 0.12 |
| SCRN1 | ENST00000242059.5 | secernin 1 | -0.22 | 0.11 |
| NOTCH2 | ENST00000256646.2 | notch 2 | -0.03 | 0.11 |
| MEGF9 | ENST00000373930.3 | multiple EGF-like-domains 9 | -0.02 | 0.11 |
| DMRTC1B | ENST00000373532.3 | DMRT-like family C1B | -0.4 | < 0.1 |
| PSRC1 | ENST00000409267.1 | proline/serine-rich coiled-coil 1 | -0.35 | < 0.1 |
| PCBD1 | ENST00000299299.3 | pterin-4 alpha-carbinolamine dehydratase/dimerization cofactor of hepatocyte nuclear factor 1 alpha | -0.32 | < 0.1 |
| KPNA4 | ENST00000334256.4 | karyopherin alpha 4 (importin alpha 3) | -0.31 | < 0.1 |
| SEC22C | ENST00000264454.3 | SEC22 vesicle trafficking protein homolog C (S. cerevisiae) | -0.29 | < 0.1 |
| EPHA4 | ENST00000281821.2 | EPH receptor A4 | -0.25 | < 0.1 |
| PPP1R3B | ENST00000310455.3 | protein phosphatase 1, regulatory subunit 3B | -0.25 | < 0.1 |
| ESYT2 | ENST00000251527.5 | extended synaptotagmin-like protein 2 | -0.21 | < 0.1 |
| POC1B | ENST00000378528.2 | POC1 centriolar protein B | -0.2 | < 0.1 |
| PURG | ENST00000475541.1 | purine-rich element binding protein G | -0.18 | < 0.1 |
| ABCB7 | ENST00000253577.3 | ATP-binding cassette, sub-family B (MDR/TAP), member 7 | -0.18 | < 0.1 |
| ACBD5 | ENST00000396271.3 | acyl-CoA binding domain containing 5 | -0.14 | < 0.1 |
| BRWD3 | ENST00000373275.4 | bromodomain and WD repeat domain containing 3 | -0.12 | < 0.1 |
| FCHO2 | ENST00000430046.2 | FCH domain only 2 | -0.11 | < 0.1 |
| TESK2 | ENST00000372084.1 | testis-specific kinase 2 | -0.09 | < 0.1 |
| UNC80 | ENST00000439458.1 | unc-80 homolog (C. elegans) | -0.03 | < 0.1 |
| RNFT1 | ENST00000442346.2 | ring finger protein, transmembrane 1 | -0.46 | ORF |
| MCAM | ENST00000392814.1 | melanoma cell adhesion molecule | -0.13 | ORF |
| PLD1 | ENST00000342215.6 | phospholipase D1, phosphatidylcholine-specific | -0.06 | ORF |
| NIPBL | ENST00000448238.2 | Nipped-B homolog (Drosophila) | -0.06 | ORF |

**Supplementary Table2.Clinical data of the ICH patients**

| **Clinical data** |  |
| --- | --- |
| Mean age±SD, y | 72.5±7.8 |
| Sex (female), % | 10 (0.5) |
| Hypertension, % | 18(0.9) |
| Diabetes mellitus, % | 5(0.25) |
| Hypercholesterolemia, % | 8(0.4) |
| Warfarin, % | 5(0.25) |
| Antiplatelets, % | 8(0.4) |
| Previous ICH Ischemic stroke,% | 1(0.5) |
| Acute coronary event,% | 2(0.1） |
| **Coagulation function test** |  |
| INR(Internationalnormalized ratio)±SD, y | 1.16±0.27 |
| APTT(s)±SD, y | 31.16±6.2 |
| Platelet count (103/μl) ±SD, y | 196±83.2 |
| **Intervention** |  |
| Surgery or Hematoma evacuation, % | 0（0） |
| Medication (n (%)) | 20（1.0） |
| **Prognosis evaluation** |  |
| NIHSS score ±SD, y | 12.05 ±5.98 |
| Volume of hematoma( ml )±SD, y | 20.12±12.34 |
| **mRS score at 90 days** |  |
| a.Rankin Scale score of 0-1,% | 13(0.65) |
| b.Rankin Scale score of 2-6 ,% | 7(0.35) |
